# Supplementary material for: Low connectivity between shallow, mesophotic and rariphotic zone benthos
Source: R Soc Open Sci. 2019 Sep 18;6(9):190958. doi: 10.1098/rsos.190958 (PMC6774966; doi:10.1098/rsos.190958)
Supplement: Supplementary Figures 1 - 3 [file rsos190958supp1.docx]

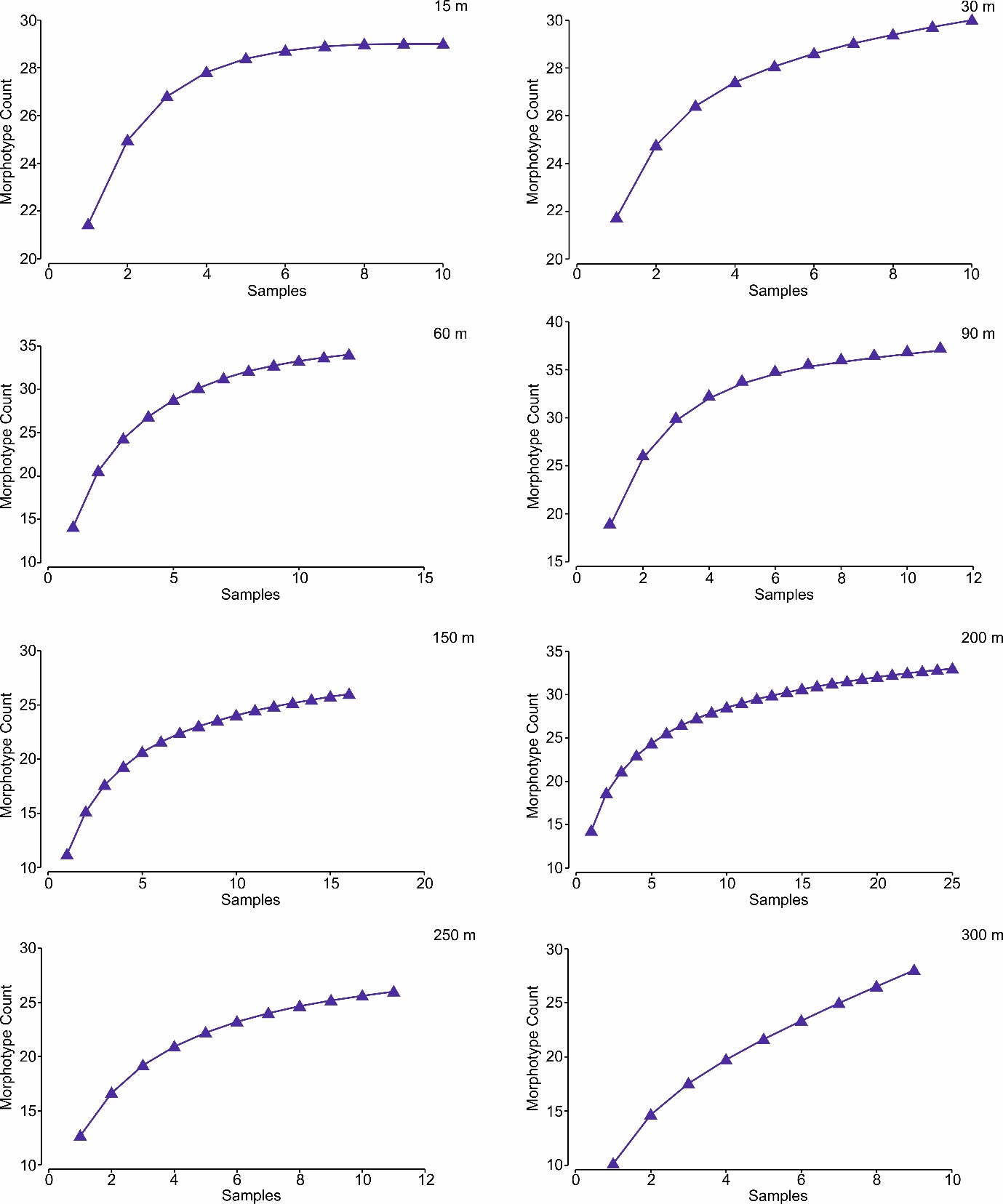


**Supplementary Fig. S1.** Morphotype accumulation curves per depth based on presence-absence data of the entire benthic community.


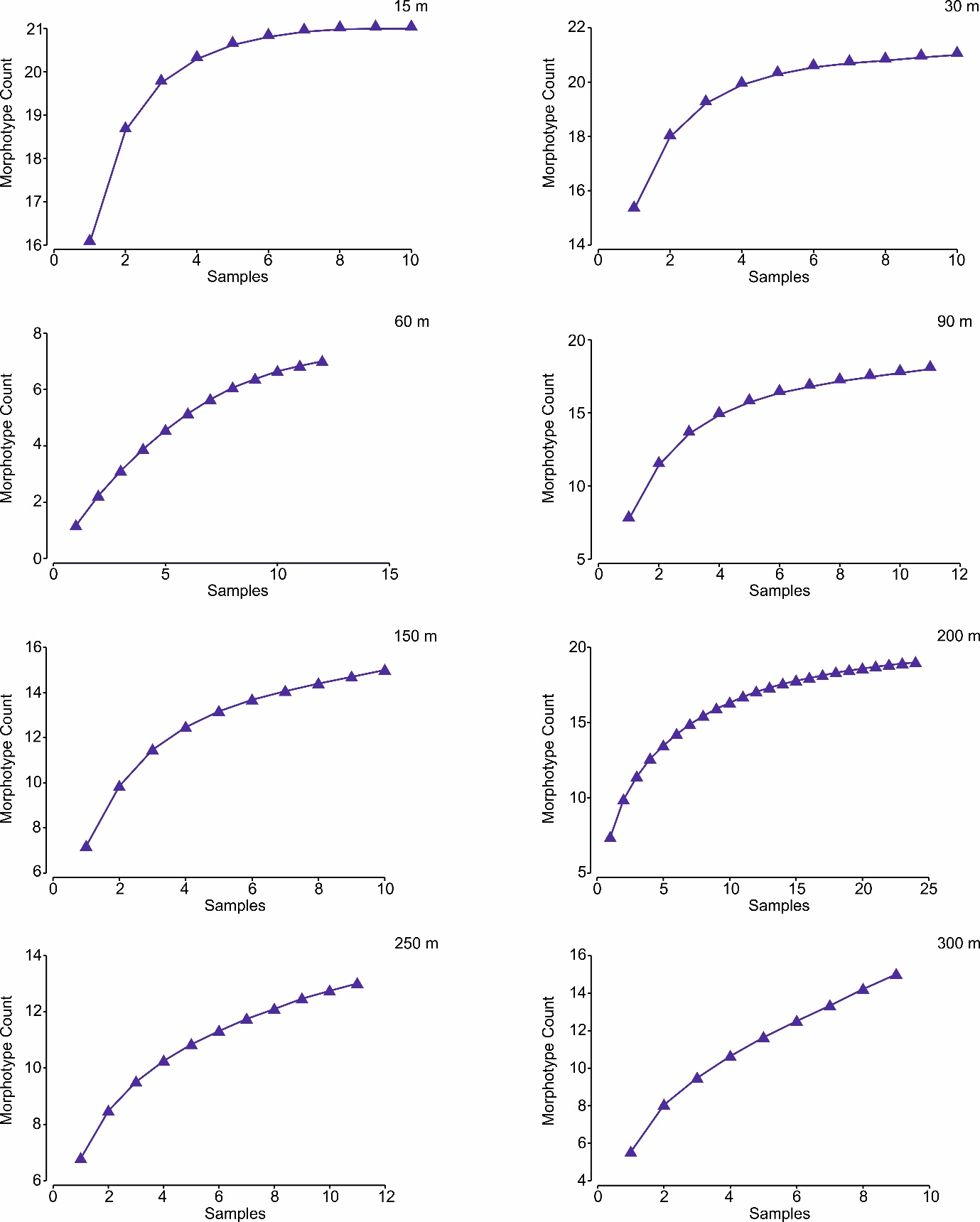


**Supplementary Fig. S2.** Morphotype accumulation curves per depth based on abundance data of numerated habitat forming taxa only (i.e. all corals and some sponges).


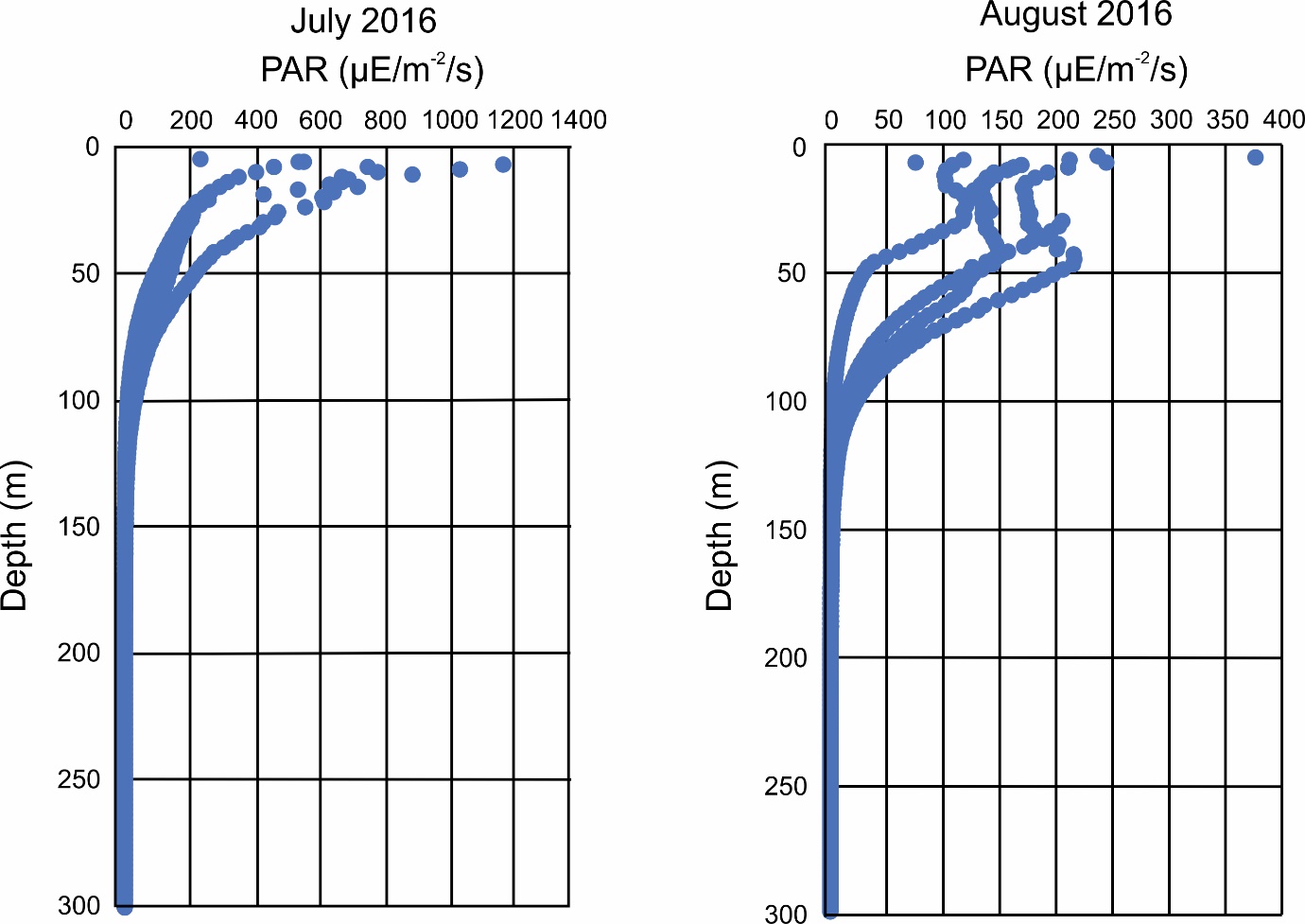


**Supplementary Fig. S3.** Vertical profiles of photosynthetically active irradiance (PAR) in Bermuda Atlantic Time-series Station. CTD casts 10326001 to 10326004 (http://batsftp.bios.edu/BATS/ctd/ASCII/b10326_ctd.txt) and 10327001 to 10327005 (http://batsftp.bios.edu/BATS/ctd/ASCII/b10327_ctd.txt),) were used to create the July and August plots, respectively.
