# Supplementary material for: Low connectivity between shallow, mesophotic and rariphotic zone benthos
Source: R Soc Open Sci. 2019 Sep 18;6(9):190958. doi: 10.1098/rsos.190958 (PMC6774966; doi:10.1098/rsos.190958)
Supplement: Supplementary Table 1 [file rsos190958supp2.docx]

| **Supplementary Table S1.** Detailed characteristics of each studied site and transect. NNE = North Northeast, PL = Plantagenet Bank, SP = Spittal, TIG = Tiger, DIV = Divers, SUB = Submersibles. | | | | | | | |
| --- | --- | --- | --- | --- | --- | --- | --- |
| **Transect** | **Site** | **Depth (m)** | **Assigned Depth (m)** | **Lat (°N)** | **Long (°W)** | **Date** | **Sampling method** |
| SP_60m_D1_T2_22_07_2016 | SP | 60.96 | 60 | 32.3202 | 64.6597 | 22.07.2016 | DIV |
| NNE_60m_D2_T2_23_07_2016 | NNE | 60 | 60 | 32.5036 | 64.6212 | 23.07.2016 | DIV |
| NNE_60m_D2_T3_23_07_2016 | NNE | 60 | 60 | 32.5035 | 64.6214 | 23.07.2016 | DIV |
| NNE_60m_D2_T4_23_07_2016 | NNE | 60 | 60 | 32.5035 | 64.6214 | 23.07.2016 | DIV |
| NNE_30m_D2_T5_23_07_2016 | NNE | 30.7 | 30 | 32.5011 | 64.6306 | 23.07.2016 | DIV |
| NNE_30m_D2_T6_23_07_2016 | NNE | 30.7 | 30 | 32.5011 | 64.6306 | 23.07.2016 | DIV |
| NNE_15m_D2_T7_23_07_2016 | NNE | 18.4-20.3 | 15 | 32.4969 | 64.6306 | 23.07.2016 | DIV |
| PL_90m_D4_T1_25_07_2016 | PL | 91.7-93.5 | 90 | 31.9449 | 65.1569 | 25.07.2016 | DIV |
| PL_90m_D4_T2_25_07_2016 | PL | 90-91.3 | 90 | 31.9449 | 65.1569 | 25.07.2016 | DIV |
| SP_90m_D5_T1_26_07_2016 | SP | 90.2 | 90 | 32.3189 | 64.6537 | 26.07.2016 | DIV |
| SP_30m_D5_T3_26_07_2016 | SP | 28-28.7 | 30 | 32.3189 | 64.6537 | 26.07.2016 | DIV |
| SP_30m_D5_T4_26_07_2016 | SP | 28.9-30 | 30 | 32.3189 | 64.6537 | 26.07.2016 | DIV |
| SP_15m_D5_T5_26_07_2016 | SP | 15.1-15.6 | 15 | 32.3189 | 64.6537 | 26.07.2016 | DIV |
| SP_15m_D5_T6_26_07_2016 | SP | 15.4 | 15 | 32.3189 | 64.6537 | 26.07.2016 | DIV |
| SP_90m_D6_T1_27_07_2016 | SP | 90.7-90.8 | 90 | 32.3197 | 64.6580 | 27.07.2016 | DIV |
| SP_90m_D6_T2_27_07_2016 | SP | 90.8-91 | 90 | 32.3197 | 64.6580 | 27.07.2016 | DIV |
| SP_30m_D6_T3_27_07_2016 | SP | 30.8-30.9 | 30 | 32.3220 | 64.6625 | 27.07.2016 | DIV |
| SP_15m_D6_T5_27_07_2016 | SP | 15.1-16 | 15 | 32.3255 | 64.6668 | 27.07.2016 | DIV |
| NNE_30m_D7_T2_28_07_2016 | NNE | 29.9 | 30 | 32.4604 | 64.6113 | 28.07.2016 | DIV |
| NNE_15m_D9_T1_30_07_2016 | NNE | 19.5-19.8 | 15 | 32.4604 | 64.6112 | 30.07.2016 | DIV |
| NNE_15m_D9_T2_30_07_2016 | NNE | 19.8 | 15 | 32.4604 | 64.6112 | 30.07.2016 | DIV |
| NNE_90m_D10_T2_31_07_2016 | NNE | 91.7-93.2 | 90 | 32.4819 | 64.5847 | 31.07.2016 | DIV |
| TIG_60m_D11_T1_01_08_2016 | TIG | 60.7-62.8 | 60 | 32.1925 | 64.9685 | 01.08.2016 | DIV |
| TIG_60m_D11_T2_01_08_2016 | TIG | 61-62 | 60 | 32.1925 | 64.9685 | 01.08.2016 | DIV |
| TIG_60m_D11_T4_01_08_2016 | TIG | 62 | 60 | 32.1925 | 64.9685 | 01.08.2016 | DIV |
| SP_60m_D13_T1_03_08_2016 | SP | 57.9 | 60 | 32.3201 | 64.6580 | 03.08.2016 | DIV |
| SP_60m_D13_T2_03_08_2016 | SP | 62.8 | 60 | 32.3201 | 64.6580 | 03.08.2016 | DIV |
| NNE_90m_D14_T1_04_08_2016 | NNE | 91.1 | 90 | 32.4782 | 64.5791 | 04.08.2016 | DIV |
| NNE_90m_D14_T2_04_08_2016 | NNE | 91.1 | 90 | 32.4782 | 64.5791 | 04.08.2016 | DIV |
| TIG_90m_D15_T1_05_08_2016 | TIG | 89.6 | 90 | 32.1936 | 64.9673 | 05.08.2016 | DIV |
| TIG_90m_D15_T2_05_08_2016 | TIG | 92.7 | 90 | 32.1936 | 64.9673 | 05.08.2016 | DIV |
| TIG_30m_D15_T3_05_08_2016 | TIG | 30.2 | 30 | 32.2067 | 64.9569 | 05.08.2016 | DIV |
| TIG_30m_D15_T4_05_08_2016 | TIG | 31.4 | 30 | 32.2067 | 64.9569 | 05.08.2016 | DIV |
| TIG_15m_D15_T5_05_08_2016 | TIG | 17 | 15 | 32.1991 | 64.9676 | 05.08.2016 | DIV |
| TIG_15m_D15_T6_05_08_2016 | TIG | 17 | 15 | 32.1991 | 65.9676 | 05.08.2016 | DIV |
| TIG_90m_D17_T1_07_08_2016 | TIG | 90.2 | 90 | 32.2036 | 64.9491 | 07.08.2016 | DIV |
| TIG_30m_D17_T3_07_08_2016 | TIG | 28-28.7 | 30 | 32.2036 | 64.9491 | 07.08.2016 | DIV |
| TIG_30m_D17_T4_07_08_2016 | TIG | 28.9-30 | 30 | 32.2036 | 64.9491 | 07.08.2016 | DIV |
| TIG_15m_D17_T5_07_08_2016 | TIG | 15.1-15.6 | 15 | 32.2036 | 64.9491 | 07.08.2016 | DIV |
| TIG_15m_D17_T6_07_08_2016 | TIG | 15.4 | 15 | 32.2036 | 64.9491 | 07.08.2016 | DIV |
| PL_60m_D20_T1_12_08_2016 | PL | 55.18 | 60 | 31.9499 | 65.1776 | 12.08.2016 | DIV |
| PL_60m_D20_T3_12_08_2016 | PL | 55.18 | 60 | 31.9499 | 65.1776 | 12.08.2016 | DIV |
| PL_60m_D20_T4_12_08_2016 | PL | 56 | 60 | 31.9499 | 65.1776 | 12.08.2016 | DIV |
| NNE_150m_D3_T1_23_07_2016 | NNE | 145-146 | 150 | 32.5024 | 64.6168 | 23.07.2016 | SUB |
| NNE_150m_D3_T2_23_07_2016 | NNE | 137-139 | 150 | 32.5000 | 64.6139 | 23.07.2016 | SUB |
| NNE_150m_D3_T3_23_07_2016 | NNE | 136-139 | 150 | 32.4973 | 64.6104 | 23.07.2016 | SUB |
| PL_200m_D4_T1_24_07_2016 | PL | 186-187 | 200 | 31.9424 | 65.1564 | 24.07.2016 | SUB |
| PL_300m_D5_T1_25_07_2016 | PL | 297-302 | 300 | 31.9395 | 65.1570 | 25.07.2016 | SUB |
| PL_300m_D5_T2_25_07_2016 | PL | 303 | 300 | 31.9375 | 65.1618 | 25.07.2016 | SUB |
| PL_300m_D5_T3_25_07_2016 | PL | 303 | 300 | 31.9355 | 65.1643 | 25.07.2016 | SUB |
| PL_150m_D6_T1_25_07_2016 | PL | 137 | 150 | 31.9453 | 65.1575 | 25.07.2016 | SUB |
| SP_300m_D7_T1_26_07_2016 | SP | 300 | 300 | 32.3165 | 64.6561 | 26.07.2016 | SUB |
| SP_300m_D7_T2_26_07_2016 | SP | 297-300 | 300 | 32.3160 | 64.6531 | 26.07.2016 | SUB |
| SP_300m_D7_T3_26_07_2016 | SP | 300-301 | 300 | 32.3113 | 64.6569 | 26.07.2016 | SUB |
| SP_200m_D8_T1_26_07_2016 | SP | 200 | 200 | 32.3202 | 64.6552 | 26.07.2016 | SUB |
| SP_200m_D8_T2_26_07_2016 | SP | 200-202 | 200 | 32.3173 | 64.6554 | 26.07.2016 | SUB |
| SP_200m_D8_T3_26_07_2016 | SP | 199-200 | 200 | 32.3142 | 64.6582 | 26.07.2016 | SUB |
| NNE_300m_D10_T1_28_07_2016 | NNE | 299-303 | 300 | 32.4851 | 64.5864 | 28.07.2016 | SUB |
| NNE_300m_D10_T2_28_07_2016 | NNE | 300-301 | 300 | 32.4841 | 64.5859 | 28.07.2016 | SUB |
| NNE_300m_D10_T3_28_07_2016 | NNE | 299-301 | 300 | 32.4842 | 64.5808 | 28.07.2016 | SUB |
| NNE_200m_D11_T1_28_07_2016 | NNE | 200 | 200 | 32.4830 | 64.5809 | 28.07.2016 | SUB |
| NNE_200m_D11_T2_28_07_2016 | NNE | 200 | 200 | 32.4804 | 64.5793 | 28.07.2016 | SUB |
| NNE_200m_D11_T3_28_07_2016 | NNE | 199 | 200 | 32.4783 | 64.5772 | 28.07.2016 | SUB |
| NNE_250m_D12_T1_29_07_2016 | NNE | 249-252 | 250 | 32.4861 | 64.5835 | 29.07.2016 | SUB |
| NNE_250m_D12_T2_29_07_2016 | NNE | 250 | 250 | 32.4816 | 64.5802 | 29.07.2016 | SUB |
| NNE_250m_D12_T3_29_07_2016 | NNE | 250 | 250 | 32.4792 | 64.5775 | 29.07.2016 | SUB |
| NNE_200m_D13_T1_31_07_2016 | NNE | 200 | 200 | 32.4840 | 64.5823 | 31.07.2016 | SUB |
| NNE_200m_D13_T2_31_07_2016 | NNE | 200 | 200 | 32.4797 | 64.5789 | 31.07.2016 | SUB |
| NNE_200m_D13_T3_31_07_2016 | NNE | 200 | 200 | 32.4766 | 64.5758 | 31.07.2016 | SUB |
| NNE_200m_D14_T1_31_07_2016 | NNE | 200 | 200 | 32.4782 | 64.5767 | 31.07.2016 | SUB |
| NNE_200m_D14_T2_31_07_2016 | NNE | 199-200 | 200 | 32.4750 | 64.5750 | 31.07.2016 | SUB |
| NNE_200m_D14_T3_31_07_2016 | NNE | 196-200 | 200 | 32.4755 | 64.5726 | 31.07.2016 | SUB |
| TIG_200m_D15_T1_01_08_2016 | TIG | 200 | 200 | 32.1909 | 64.9684 | 01.08.2016 | SUB |
| TIG_200m_D15_T2_01_08_2016 | TIG | 200 | 200 | 32.1936 | 64.9684 | 01.08.2016 | SUB |
| TIG_200m_D15_T3_01_08_2016 | TIG | 199 | 200 | 32.1897 | 64.9699 | 01.08.2016 | SUB |
| TIG_150m_D15_T4_01_08_2016 | TIG | 137-141 | 150 | 32.1881 | 64.9702 | 01.08.2016 | SUB |
| TIG_150m_D15_T5_01_08_2016 | TIG | 137-142 | 150 | 32.1867 | 64.9705 | 01.08.2016 | SUB |
| TIG_150m_D15_T6_01_08_2016 | TIG | 140-142 | 150 | 32.1859 | 64.9717 | 01.08.2016 | SUB |
| SP_150m_D18_T1_04_08_2016 | SP | 149-150 | 150 | 32.3221 | 64.6563 | 04.08.2016 | SUB |
| SP_150m_D18_T2_04_08_2016 | SP | 148-149 | 150 | 32.3153 | 64.6594 | 04.08.2016 | SUB |
| SP_150m_D18_T3_04_08_2016 | SP | 149-150 | 150 | 32.3130 | 64.6597 | 04.08.2016 | SUB |
| SP_250m_D19_T1_04_08_2016 | SP | 250 | 250 | 32.3204 | 64.6547 | 04.08.2016 | SUB |
| SP_250m_D19_T2_04_08_2016 | SP | 248-251 | 250 | 32.3209 | 64.6523 | 04.08.2016 | SUB |
| SP_250m_D19_T3_04_08_2016 | SP | 249-250 | 250 | 32.3137 | 64.6511 | 04.08.2016 | SUB |
| SP_150m_D19_T4_04_08_2016 | SP | 143-148 | 150 | 32.3153 | 64.6598 | 04.08.2016 | SUB |
| TIG_250m_D20_T1_05_08_2016 | TIG | 241 | 250 | 32.1920 | 64.9669 | 05.08.2016 | SUB |
| TIG_250m_D20_T2_05_08_2016 | TIG | 243 | 250 | 32.1912 | 64.9696 | 05.08.2016 | SUB |
| NNE_200m_D21_T1_06_08_2016 | NNE | 195-200 | 200 | 32.4765 | 64.5744 | 06.08.2016 | SUB |
| NNE_200m_D21_T2_06_08_2016 | NNE | 198-200 | 200 | 32.4743 | 64.5759 | 06.08.2016 | SUB |
| NNE_200m_D21_T3_06_08_2016 | NNE | 147-151 | 200 | 32.4730 | 64.5749 | 06.08.2016 | SUB |
| SP_150m_D22_T1_07_08_2016 | SP | 148-153 | 150 | 32.3157 | 64.6568 | 07.08.2016 | SUB |
| SP_150m_D22_T2_07_08_2016 | SP | 148-150 | 150 | 32.3164 | 64.6581 | 07.08.2016 | SUB |
| SP_150m_D22_T3_07_08_2016 | SP | 147-151 | 150 | 32.3166 | 64.6584 | 07.08.2016 | SUB |
| TIG_200m_D26_T4_12_08_2016 | TIG | 198-200 | 200 | 32.1791 | 64.9865 | 12.08.2016 | SUB |
| TIG_200m_D27_T1_12_08_2016 | TIG | 200 | 200 | 32.1802 | 64.9812 | 12.08.2016 | SUB |
| TIG_200m_D27_T2_12_08_2016 | TIG | 200 | 200 | 32.1811 | 64.9837 | 12.08.2016 | SUB |
| PL_250m_D28_T1_13_08_2016 | PL | 249 | 250 | 31.9424 | 65.1549 | 13.08.2016 | SUB |
| PL_250m_D28_T2_13_08_2016 | PL | 250 | 250 | 31.9400 | 65.1590 | 13.08.2016 | SUB |
| PL_250m_D28_T3_13_08_2016 | PL | 249 | 250 | 31.9404 | 65.1628 | 13.08.2016 | SUB |
| PL_200m_D28_T4_13_08_2016 | PL | 198-199 | 200 | 31.9387 | 65.1648 | 13.08.2016 | SUB |
| PL_200m_D29_T1_13_08_2016 | PL | 200-202 | 200 | 31.9424 | 65.1549 | 13.08.2016 | SUB |
| PL_200m_D29_T2_13_08_2016 | PL | 197-199 | 200 | 31.9400 | 65.1590 | 13.08.2016 | SUB |
| PL_150m_D29_T3_13_08_2016 | PL | 148-150 | 150 | 31.9404 | 65.1628 | 13.08.2016 | SUB |
| PL_150m_D29_T4_13_08_2016 | PL | 146-151 | 150 | 31.9387 | 65.1648 | 13.08.2016 | SUB |
