# Supplementary material for: Low connectivity between shallow, mesophotic and rariphotic zone benthos
Source: R Soc Open Sci. 2019 Sep 18;6(9):190958. doi: 10.1098/rsos.190958 (PMC6774966; doi:10.1098/rsos.190958)
Supplement: Supplementary Table 2 [file rsos190958supp3.docx]

| **SupplementaryTable S2.** Changes of benthic community structure in relation to depth and site using PERMANOVA on Bray-Curtis similarity matrices of presence-absence data (entire benthic community) and log(x+1)-transformed abundance data (all corals and numerated sponges). Only significant pairwise comparisons (p ≤ 0.05) are shown. NNE = North Northeast, PL = Plantagenet Bank, SP = Spittal, TIG = Tiger. | | | | | | |
| --- | --- | --- | --- | --- | --- | --- |
|  | **Entire benthic community** | | | **Corals and Sponges** | | |
|  | **Pseudo-F** | **P(perm)** | **Unique perms** | **Pseudo-F** | **P(perm)** | **Unique perms** |
| Depth | 54.826 | <0.001 | 9885 | 45.913 | <0.001 | 9892 |
| Site | 4.7233 | <0.001 | 9919 | 3.6904 | <0.001 | 9941 |
| Depth x Site | 3.5093 | <0.001 | 9848 | 3.9962 | <0.001 | 9838 |
|  |  |  |  |  |  |  |
| **Pairwise comparisons against Depth (m)** | **t** | **P(perm)** | **Unique perms** | **t** | **P(perm)** | **Unique perms** |
| 15, 30 | 3.3008 | <0.001 | 9963 | 3.9373 | <0.001 | 9954 |
| 15, 60 | 7.5407 | <0.001 | 9940 | 4.6199 | <0.001 | 9955 |
| 15, 90 | 9.5077 | <0.001 | 9946 | 8.2615 | <0.001 | 9934 |
| 15, 150 | 7.931 | <0.001 | 9926 | 7.952 | <0.001 | 9936 |
| 15, 200 | 9.9941 | <0.001 | 9934 | 12.702 | <0.001 | 9941 |
| 15, 250 | 10.891 | <0.001 | 9941 | 18.034 | <0.001 | 9933 |
| 15, 300 | 8.9882 | <0.001 | 9953 | 8.8901 | <0.001 | 9937 |
| 30, 60 | 7.0716 | <0.001 | 9953 | 4.3541 | <0.001 | 9939 |
| 30, 90 | 9.2496 | <0.001 | 9937 | 7.5888 | <0.001 | 9944 |
| 30, 150 | 7.8415 | <0.001 | 9940 | 7.0595 | <0.001 | 9955 |
| 30, 200 | 9.7261 | <0.001 | 9940 | 11.485 | <0.001 | 9944 |
| 30, 250 | 10.897 | <0.001 | 9930 | 13.464 | <0.001 | 9931 |
| 30, 300 | 9.2044 | <0.001 | 9952 | 7.8997 | <0.001 | 9933 |
| 60, 90 | 3.7257 | <0.001 | 9957 | 3.3038 | <0.001 | 9949 |
| 60, 150 | 5.4904 | <0.001 | 9942 | 3.2349 | <0.001 | 9940 |
| 60, 200 | 7.7779 | <0.001 | 9942 | 5.7543 | <0.001 | 9930 |
| 60, 250 | 7.5208 | <0.001 | 9938 | 5.183 | <0.001 | 9930 |
| 60, 300 | 6.9694 | <0.001 | 9936 | 3.5814 | <0.001 | 9929 |
| 90, 150 | 4.8614 | <0.001 | 9942 | 4.4298 | <0.001 | 9944 |
| 90, 200 | 7.3109 | <0.001 | 9932 | 8.2042 | <0.001 | 9943 |
| 90, 250 | 7.6397 | <0.001 | 9938 | 7.8775 | <0.001 | 9931 |
| 90, 300 | 7.0699 | <0.001 | 9936 | 5.5028 | <0.001 | 9938 |
| 150, 200 | 3.017 | <0.001 | 9951 | 3.8327 | <0.001 | 9947 |
| 150, 250 | 3.7701 | <0.001 | 9939 | 4.9248 | <0.001 | 9953 |
| 150, 300 | 4.4304 | <0.001 | 9949 | 2.9865 | <0.001 | 9943 |
| 200, 250 | 1.3983 | 0.067 | 9947 | 1.7324 | 0.033 | 9950 |
| 200, 300 | 3.1417 | <0.001 | 9947 | 1.1088 | 0.3 | 9927 |
| 250, 300 | 2.4642 | 0.001 | 9938 | 1.4124 | 0.063 | 9946 |
|  |  |  |  |  |  |  |
| **Pairwise comparisons against Site** |  |  |  |  |  |  |
| **15‒30 m** | **t** | **P(perm)** | **Unique perms** | **t** | **P(perm)** | **Unique perms** |
| NNE, SP | 0.99236 | 0.393 | 8938 | 1.1885 | 0.231 | 8960 |
| NNE, TIG | 0.79053 | 0.647 | 9912 | 1.2659 | 0.169 | 9896 |
| SP, TIG | 1.7303 | 0.051 | 9911 | 1.1471 | 0.258 | 9910 |
|  |  |  |  |  |  |  |
| **60‒90 m** |  |  |  |  |  |  |
| SP, NNE | 2.4683 | 0.004 | 8916 | 2.2635 | 0.001 | 471 |
| SP, PL | 2.2405 | <0.001 | 9003 | 1.3192 | 0.155 | 4337 |
| SP, TIG | 2.6644 | <0.001 | 8917 | 2.3303 | 0.002 | 5809 |
| NNE, PL | 2.847 | <0.001 | 8880 | 1.8899 | 0.01 | 1120 |
| NNE, TIG | 3.506 | <0.001 | 8956 | 2.5539 | 0.001 | 4650 |
| PL, TIG | 2.2302 | <0.001 | 8941 | 1.8848 | 0.003 | 8854 |
|  |  |  |  |  |  |  |
| **150‒300 m** |  |  |  |  |  |  |
| NNE, PL | 2.2025 | 0.003 | 9950 | 2.0329 | 0.006 | 9949 |
| NNE, SP | 1.8918 | 0.01 | 9942 | 1.6049 | 0.056 | 9931 |
| NNE, TIG | 1.6844 | 0.03 | 9944 | 1.1498 | 0.26 | 9954 |
| PL, SP | 2.5404 | 0.001 | 9950 | 1.5785 | 0.071 | 9947 |
| PL, TIG | 1.8801 | 0.014 | 9927 | 2.1646 | 0.003 | 9931 |
| SP, TIG | 1.0315 | 0.36 | 9950 | 2.0099 | 0.012 | 9942 |
