# Supplementary material for: Low connectivity between shallow, mesophotic and rariphotic zone benthos
Source: R Soc Open Sci. 2019 Sep 18;6(9):190958. doi: 10.1098/rsos.190958 (PMC6774966; doi:10.1098/rsos.190958)
Supplement: Supplementary Table 3 [file rsos190958supp4.docx]

| **Supplementary Table S3.** Percentage of variation explained by benthic cover and substratum composition in a distance-based multilinear model for megabenthic communities using presence-absence (P-A) and log(x+1)-transformed abundance (N) data. | | | | | |
| --- | --- | --- | --- | --- | --- |
| **Dependent variable** | | **Explanatory variable** | **Pseudo-*F*** | ***P*** | **%Variation explained** |
| **P-A** |  | Depth | 58.534 | <0.001 | 39.68 |
|  |  | Bedrock | 6.917 | <0.001 | 7.21 |
|  |  | Boulders | 0.746 | 0.6604 | 0.83 |
|  |  | Rhodoliths | 3.848 | 0.008 | 4.14 |
|  |  | Sediment | 8.108 | <0.001 | 8.35 |
|  | BEST model | Depth + Bedrock + Rhodoliths + Sediment |  |  | 50.66 |
|  |  |  |  |  |  |
| **N** |  | Depth | 49.643 | <0.001 | 36.87 |
|  |  | Bedrock | 6.172 | <0.001 | 6.77 |
|  |  | Boulders | 0.493 | 0.988 | 0.58 |
|  |  | Rhodoliths | 3.956 | 0.006 | 4.45 |
|  |  | Sediment | 6.766 | <0.001 | 7.37 |
|  | BEST model | Depth + Bedrock + Rhodoliths + Sediment |  |  | 44.95 |
