# Supplementary material for: Low connectivity between shallow, mesophotic and rariphotic zone benthos
Source: R Soc Open Sci. 2019 Sep 18;6(9):190958. doi: 10.1098/rsos.190958 (PMC6774966; doi:10.1098/rsos.190958)
Supplement: Supplementary Material 1 [file rsos190958supp5.docx]

**S1 Material. Detailed survey design.**

We examined reef habitats between 15 m and 305 m depth at three sites: North Northeast, Spittal, and Tiger, along the north east, south east and southern slopes of the Bermuda platform respectively, and on one adjacent seamount (Plantagenet Bank). All transect surveys were conducted during 17 July ‒ 14 August 2016 on board the *R/V Baseline Explorer*. Research permits for Bermuda were issued by the Department of Environment and Natural Resources, Bermuda (Ship Approval No. 87/2016).

Data from shallowest transects (15‒94 m depth) were gathered by a team of nine technical divers (Global Underwater Explorers; GUE) equipped with closed-circuit rebreathers (JJ-CCR CE Edition [JJ-CCR ApS, Copenhagen, Denmark] modified to GUE Configuration, GUE, High Springs, Florida, USA – see [<https://www.divegue.tv/programs/gue-rebreather-diver-jj-ccr-overview?autoplay=true>]) and diver propulsion vehicles. Transects were filmed using a diver-operated stereo-video system (SeaGIS, Melbourne, Australia) consisting of two cameras (GoPro Hero 4 Camera in custom-built SeaGIS housing) and two lights, pointed at an angle of 3^o^ and spaced 80 cm apart. The transects followed a 50 m survey tape laid along the depth contour, with the cameras facing forward, about 1.5 m off the seafloor, in order to record reef fish assemblages [1]. The same survey tapes were also used to guide video transects that recorded the substratum type / composition and the diversity and density of life on the seafloor (benthos), using the camera and light system oriented perpendicular to the seabed. Transects were ~6 min long. In total, 43 benthic transects were conducted by technical divers at ~15 m (10 transects), 30 m (10), 60 m (12) and 90 m (11).

Videos of the deeper transects (136‒305 m) were collected using the *Nemo* and *Nomad* Triton 1000-2 class submersibles (Triton submersibles, Vero Beach, Florida, USA). The *Nemo* submersible primarily carried out video surveys, whilst *Nomad* was employed for water and biological sampling, and for high-resolution filming of operations and marine life. *Nemo* was equipped with four forward facing Deep-Sea Matrix-1 LED Lights to illuminate the water column and the seafloor, a submersible-operated stereo-video system (for the fish assemblage surveys [1]) comprising two cameras (GoPro Hero 4 Camera in Scout Pro Go Benthic 2 1000 m depth-rated housing; Group Binc, Jensen Beach, Florida, USA) mounted on the lower starboard nacelle along an aluminium bar, pointed at an angle of 8^o^ and spaced 80 cm apart, and a downward-pointing camera (for the benthic surveys) equipped with Teledyne-Bowtech Ocean Lasers (Green) spaced at 25 cm, and two lights. Submersible position and depth was measured via a TrackLink 1500 USBL acoustic tracking system (LinkQuest Inc., San Diego, California, USA), which was calibrated with PneumoCal Pneumo Gauges. The submersible pilot aimed to maintain a consistent distance from the substrate during surveys, but this was not always possible as terrain was of varying steepness and topography. Transects consisted of 20 min of filming, and covered an estimated distance of 100 m at a speed of 0.2 knots. A 5-min gap, during which the submersible traversed the seabed, was used to separate replicate transects at a given depth. In total, 61 benthic transects were conducted by submersibles at ~150 m (16 transects), 200 m (25), 250 m (11), and 300 m (9). Detailed characteristics of each transect survey can be found in electronic supplementary table, S1.

Conductivity, temperature and salinity were measured down to depths of 94 m by the technical divers using a YSI EXO1 Multiparameter Sonde (YSI Incorporated, Yellow Springs, Ohio, USA). The submersible *Nomad* was fitted with a SBE 49 FastCAT CTD Sensor (SeaBird Electronics Inc., Bellevue, Washington, USA) to measure the same parameters between 136‒305 m.

**References**

1. Stefanoudis P.V., Gress E., Pitt J.M., Smith S.R., Kincaid T., Rivers M., Andradi-Brown D., Rowlands G., Woodall L.C., Rogers A.D. 2019 Depth-dependent structuring of reef fish assemblanges from the shallows to the rariphotic zone. *Frontiers in Marine Science* **6**, 307. (doi:10.3389/fmars.2019.00307).
